# Is the combination of depression symptoms and multimorbidity associated with the increase of the prevalence of functional disabilities in Brazilian older adults? A cross-sectional study

**DOI:** 10.3389/fragi.2023.1188552

**Published:** 2023-05-23

**Authors:** Patrícia Pereira de Souza da Rosa, Larissa Pruner Marques, Vanessa Pereira Corrêa, Cesar De Oliveira, Ione Jayce Ceola Schneider

**Affiliations:** ^1^ Graduate Program in Rehabilitation Science, Federal University of Santa Catarina, Araranguá, Brazil; ^2^ Oswaldo Cruz Foundation, Rio de Janeiro, Brazil; ^3^ Graduate Program in Collective Health, Federal University of Santa Catarina, Florianópolis, Brazil; ^4^ Epidemiology and Public Health Department, University College London, London, United Kingdom

**Keywords:** older adult, multimorbidity, activities of daily living, depressive symptoms, prevalence

## Abstract

**Introduction:** Functional disabilities are more prevalent in older adults with multimorbidity and depression. However, few studies have investigated the combination of multimorbidity and depression with functional disability. This study aims to verify whether symptoms of depression and multimorbidity combined increase the prevalence of functional disability in Brazilian older adults.

**Material and methods:** This is a cross-sectional study conducted with data from the Brazilian Longitudinal Study of Aging (ELSI-Brazil) baseline examination in 2015–2016 in adults aged 50 years and older. The variables included were basic (BADL) and instrumental activities of daily living (IADL), depressive symptoms, multimorbidity (≥2 chronic diseases), sociodemographic variables, and lifestyle. Logistic regression was performed to estimate crude and adjusted odds ratios.

**Results:** A total of 7,842 participants over 50 years of age were included. Of these, 53.5% were women and 50.5% were between 50 and 59 years old, 33.5% reported ≥4 depressive symptoms, 51.4% had multimorbidity, 13.5% reported difficulty in performing at least one BADL, and 45.1% reported difficulty in performing the IADL. In the adjusted analysis, the prevalence of difficulty on BADL was 6.52 (95% CI: 5.14; 8.27) and on IADL was 2.34 (95% CI: 2.15; 2.55), higher for those with depression and multimorbidity combined when compared with those without these conditions.

**Conclusion:** The combination of symptoms of depression and multimorbidity may increase functional impairments in the BADL and IADL of Brazilian older adults, impairing self-efficacy, independence, and autonomy. Early detection of these factors benefits the person, their family, and the healthcare system for health promotion and disease prevention.

## 1 Introduction

Aging is considered a worldwide social phenomenon and a challenge for humanity. The forecast for 2050 is approximately two billion people aged 60 years or more worldwide. In Brazil, there were 14.1 million older adults in 2002, but this has been estimated to reach 33.4 million in 2025 (World Health Organization, 2005). The increase in longevity has led to several changes in the health profile of the population, which include an increase in chronic non-communicable diseases (World Health Organization, 2021).

Depression is among the most common chronic diseases in older adults and, in general, among the five most prevalent in this age group, but it still remains underdiagnosed (Sadock et al., 2017). The Pan American Health Organization (2018) considers depression to be one of the main causes of disability. It generates abnormal behaviors, affects interpersonal relationships, and results in feelings, thoughts, and perceptions outside healthy standards (Pan American Health Organization, 2018).

Some studies emphasize the association of mental disorders with multimorbidity (Prior et al., 1832; Fässberg et al., 2016; Balázs et al., 2018; Lee et al., 2018; Quiñones et al., 2018). Multimorbidity is the existence of two or more chronic diseases simultaneously in an individual (Mundial de Saúde, 2015). It is a condition that generates greater use of health services (Souza et al., 2019) and affects a person’s functionality to perform the basic activities of daily living (BADL), impairs self-efficacy, and generates greater dependence on care (Peters et al., 2019). Disabilities are present in people with frailty, multimorbidity, and mental illness. Thus, new public policies directed to multimorbidity are required (Garin et al., 2014; Forjaz et al., 2015; DiNapoli et al., 2016; Lee et al., 2018).

Although older adults are in the age group most affected by multimorbidity and disability, as well as the presence of depressive symptoms (Moussavi et al., 2007; Leles da Costa Dias et al., 2019; Uchoa et al., 2019), some studies (Forjaz et al., 2015; Rivera-Almaraz et al., 2018; Sheridan et al., 2019; St John et al., 2019) present how multimorbidity generates disabilities and chronic conditions in mental health. However, few studies start from the opposite: mental illness associated with multimorbidity as generating functional disabilities (Bruffaerts et al., 2012; DiNapoli et al., 2016). Given this problem, and the considerable number of individuals with chronic conditions and mental problems, this study aimed to verify whether symptoms of depression and multimorbidity combined increase the prevalence of functional disability in Brazilian older adults.

## 2 Materials and methods

This is a cross-sectional study with data from the baseline of the Brazilian Longitudinal Study of Aging (ELSI-Brazil), a longitudinal, population-based study, representative of the non-institutionalized Brazilian population aged 50 years or more. This study is an initiative coordinated by the Oswaldo Cruz Foundation–Minas Gerais and the Federal University of Minas Gerais. The baseline survey was carried out between 2015 and 2016, in 70 municipalities in 5 Brazilian regions (Lima-Costa et al., 2018).

The present study included participants of the ELSI-Brazil aged 50 years or older of both sexes and excluded participants with incomplete data among the variables of interest.

The exposure variables were depressive symptoms and multimorbidity. The tool for screening depressive symptoms that was chosen for this study was the Center for Epidemiological Scale–Depression (CES-D) in the short version with eight items (CES-D8). The assessment relates to symptoms reported most of the week before the interview, and the eight items that were assessed are part of three factors: depressed affect, positive affect, and somatic items (Radloff, 1977). The cutoff point was established in four or more depressive symptoms (Demakakos et al., 2014).

The presence of multimorbidity was considered from the presence of two or more chronic diseases (Nunes et al., 2019): endocrine disease (diabetes mellitus); cardiovascular diseases (systemic arterial hypertension, heart attack, angina, and heart failure); degenerative diseases (stroke, Parkinson’s disease, Alzheimer’s disease); pulmonary diseases (asthma, emphysema, chronic bronchitis, chronic obstructive pulmonary disease); osteoarticular diseases [rheumatism, osteoporosis, chronic back problem (back pain, neck pain, low back pain, sciatic pain, vertebral, or disc problems)]; cancer; and kidney disease (chronic kidney failure). All were assessed through the question “Did any doctor tell you that you have…?,” in the interviewee’s questionnaire.

From the combination of the variables of depressive symptoms and multimorbidity, four categories of participants were created: absence of depressive symptoms and multimorbidity, presence of depressive symptoms only, presence of multimorbidity only, and presence of depressive symptoms and multimorbidity.

The dependent variables were disabilities in basic (BADL) and instrumental activities of daily living (IADL). The Katz Index was used to assess the BADL in older adults. This is a functional assessment tool that makes it possible to measure an individual’s ability to perform their daily activities independently or not (Katz et al., 1970). The general performance of six functions was assessed: bathing, dressing, toileting, transference, continence, and feeding. Individuals who reported difficulty in performing at least one of the six BADL measurements were considered dependent and those who reported not having difficulties as independent (Andrade et al., 2019; Giacomin et al., 2019).

To assess the IADL, the Lawton scale was used. This scale investigates the following aspects: using modes of transportation, responsibility for own medication, shopping, housekeeping, ability to use the telephone, food preparation, and ability to handle finances. Individuals who reported difficulty in performing at least one of the IADL measurements were considered dependent (Ćwirlej-Sozańska et al., 2019; Gontijo Guerra et al., 2020).

The independent variables were sociodemographic conditions, such as age group (50–59 years, 60–69 years, 70–79 years, and 80 years and older), sex (female and male), marital status (with a partner and without a partner), color/race (white, brown, black, yellow, and indigenous), and education in years of study (no formal education, 1–8 years, 9–11 years, and 12 years or more). Life habits were analyzed by smoking habits, alcohol consumption, consumption of fruits and vegetables, physical activities performed in the week before the interview, and cognitive function.

Smoking habits were verified by questions about cigarette smoking, identifying whether the participant smoked, if they have smoked, or for how long they had quit smoking. The individuals were classified as never smoked, former smokers, and current smokers. Regarding the consumption of alcoholic beverages, the patterns were established by using the National Institute on Alcohol Abuse and Alcoholism (NIAAA) screening with the reference values: light/moderate consumption (between 1 and 7 doses/week for women, and 1 and 14 doses/week for men) and risk consumption (more than 7 doses/week for women and more than 14 doses/week for men) (Noronha et al., 2019).

Food care was verified through questions about the consumption of fruits and vegetables. It was considered adequate to consume vegetables twice or more per day for at least 5 days; to consume fruits three times or more per day for at least 5 days, and to consume two servings of vegetables and three servings of fruits for at least 5 days a week. Consumption of less than five servings per day in less than 5 days per week was considered inadequate (World Health Organization, 2003).

Physical activity was analyzed using a reduced version of the *International Physical Activity Questionnaire* (IPAQ). Older individuals who performed ≥150 min of weekly activities were considered active and insufficiently active individuals who totaled <150 min per week of these activities (Mazo and Benedetti, 2010).

Cognitive function was included because content, temporal, and prospective memories affect the functionality of an individual, as well as depression leads to cognitive alterations, among others. It was assessed through temporal orientation, memory, and verbal fluency. Temporal orientation was assessed by four questions of the Mini-Mental State Examination, which involved the day, month, year, and day of the week (Almeida, 1998). These questions were characterized as all correct answers or at least one incorrect answer. The immediate and delayed memory were assessed by reading 10 words. At the end of the reading, the individual should repeat a maximum of the words that were remembered. After 5 minutes, the interviewee was asked to repeat the same words (Da Saúde and Cruz, 2015). It was scored by adding the number of words recalled combining the delayed and immediate memory (Castro-Costa et al., 2018). This score was categorized into tertiles. Verbal fluency was assessed through the number of names of animals that the participant recalled for 1 minute (Da Saúde and Cruz, 2015), and the total number of names were classified into tertiles.

A full case analysis was performed. The descriptive analysis used absolute and relative frequencies of all study variables, with the respective confidence intervals (95% CI). To estimate the occurrence prevalence of the outcomes and the 95% CI, according to the other variables, bivariate analysis with the chi-square test (χ^2^) was used. Analyses to estimate the odds of occurrence of the outcomes according to the main exposures (multimorbidity, depressive symptoms, and combination of the two) were performed by crude and adjusted logistic regression, estimating the odds ratios with the respective 95% CI. The analyses were adjusted for sociodemographic variables (sex, age, color/race, marital status, and education), lifestyle (smoking, alcohol consumption, consumption of fruits and vegetables, and physical activity), and cognition. All analyses considered the sample weights using the *svy* command and were performed in the statistical package Stata SE version 16.

The ELSI-Brazil was approved by the Research Ethics Committee of the Oswaldo Cruz Foundation (FIOCRUZ), Minas Gerais, with the Certificate of Presentation for Ethical Consideration (CAAE) number 34.649.814.0000.5091. All participants interviewed in the study consented by signing an informed consent form to participate in the study.

## 3 Results

Of the total of 9,412 participants from ELSI-Brazil, 7,659 participants with complete data are included. There were no differential losses despite the exclusion of 1,753 individuals from the analyses due to some missing information.

In this study, 53.4% were women; the prevalent age group was 50–59 years (50.9%), followed by 60–69 years (30%); the most identified races were brown (45.0%) and white (42.0%); the majority had a partner (65.6%); and the duration was 1–4 years of study (36.7%) (Table 1).

**TABLE 1:** Descriptive analysis of the variables of the population included in the study, ELSI-Brazil, 2015–2016.

Variable	n	% (95% CI)
Sex		
Female	4,286	53.4 (50.3; 56.5)
Male	3,373	46.6 (43.5; 49.7)
Age group		
50–59 years	3,496	50.9 (46.7; 55.1)
60–69 years	2,398	30.0 (28.0; 32.1)
70–79 years	1,340	14.4 (12.5; 16.6)
80 years and more	425	4.6 (3.8; 5.5)
Marital status		
With a partner	4,576	65.6 (63.0; 68.1)
Without a partner	3,083	34.4 (31.9; 37.0)
Color/race		
White	2,980	42.1 (37.1; 47.3)
Black	766	9.8 (8.1; 11.8)
Brown	3,646	45.0 (41.0; 49.1)
Yellow	79	1.1 (0.8; 1.4)
Indigenous	188	1.9 (1.4; 2.6)
Education		
12 or more	504	7.2 (6.1; 8.5)
9–11 years	1,590	22.7 (20.9; 24.7)
5–8 years	1,607	22.3 (20.4; 24.2)
1–4 years	2,895	36.7 (34.3; 39.1)
No formal education	1,063	11.0 (8.9; 13.4)
Smoking		
Never smoked	3,455	45.2 (43.5; 46.9)
Former smoker	2,886	37.3 (35.4; 39.3)
Smokes currently	1,318	17.4 (16.0; 18.9)
Alcohol consumption		
Does not consume	6,275	79.7 (77.2; 82.1)
Light/moderate consumption	693	10.5 (8.7; 12.8)
Risk consumption	691	9.6 (8.6; 10.7)
Consumption of fruits and vegetables		
Adequate	615	8.8 (7.7; 10.0)
Inadequate	7,044	91.2 (90.0; 92.3)
Physical activity		
Active	5,227	68.9 (66.5; 71.1)
Insufficiently active	2,432	31.1 (28.8; 33.5)
Temporal orientation		
All correct	5,317	70.9 (69.0; 72.8)
At least one incorrect	2,342	29.1 (27.2; 31.0)
Verbal fluency		
Lower tertile	2,901	35.7 (32.9; 38.6)
Intermediate tertile	2,159	28.2 (26.9; 29.6)
Upper tertile	2,599	36.1 (33.3; 38.9)
Memory score		
Lower tertile	3,162	38.5 (35.8; 41.2)
Intermediate tertile	1,835	24.5 (23.1; 26.0)
Upper tertile	2,662	37.0 (34.9; 39.1)
Difficulties in basic activities of daily living		
No difficulty to perform	6,582	86.6 (85.2; 87.8)
Difficulty in performing one or more	1,077	13.4 (12.1; 14.8)
Difficulties in instrumental activities of daily living		
No difficulty to perform	3,939	54.9 (51.7; 58.0)
Difficulty in performing one or more	3,720	45.1 (42.0; 48.3)
Depressive symptoms		
Absence (≤3 symptoms)	5,007	66.7 (64.9; 68.4)
Presence (≥4 symptoms)	2,652	33.3 (31.6; 35.1)
Multimorbidity		
Absence (≤1 disease)	6,188	81.1 (79.3; 82.7)
Presence (≥2 diseases)	1,471	18.9 (17.3; 20.7)
Multimorbidity and depression		
No depressive symptoms and no multimorbidity	4,290	57.3 (55.5; 59.1)
Depressive symptoms	1,898	23.8 (22.1; 25.5)
Multimorbidity	717	9.4 (8.4; 10.5)
With depressive symptoms and multimorbidity	754	9.5 (8.6; 10.6)
Cardiovascular diseases		
No	6,715	87.8 (86.5; 89.0)
Yes	944	12.1 (10.9; 13.5)
Degenerative diseases		
No	7,256	95.0 (94.3; 95.7)
Yes	403	5.0 (4.3; 5.7)
Pulmonary disorders		
No	7,023	91.5 (90.5; 92.3)
Yes	636	8.5 (7.6; 9.5)
Osteoarticular diseases		
No	3,718	49.4 (47.1; 51.6)
Yes	3,941	50.6 (48.4; 52.8)
Cancer		
No	7,259	94.7 (93.9; 95.4)
Yes	400	5.2 (4.5; 6.0)
Kidney disease		
No	7,316	95.6 (94.9; 96.2)
Yes	343	4.4 (3.8; 5.1)

Among lifestyle habits, 45.2% reported that they never smoked, most (79.7%) did not consume alcohol, 91.2% reported consuming fruits and vegetables inadequately, and 68.9% reported being physically active. Difficulty in at least one BADL was reported by 13.4% and in IADL by 45.1%. Depressive symptoms were identified in 33.3% of the participants and multimorbidity in 18.9%. Osteoarticular (50.6%) and cardiovascular (12.1%) diseases were the most prevalent in the multimorbidity groupings. In the combination of multimorbidity and depressive symptoms, 57.0% had neither symptom while 9.5% had both symptoms (Table 1).

### 3.1 BADL

The results of the bivariate analyses of BADL by sociodemographic and health factors, depression, and multimorbidity are presented in Table 2. Difficulties in performing the BADL were associated in the bivariate analysis with increased odds of the outcomes in those aged 80 years or more, without a partner, without a formal study, who were former smokers, physically inactive, with cognitive difficulty in temporal orientation, with depressive symptoms, and with multimorbidity.

**TABLE 2:** Bivariate analysis of difficulties in basic activities of daily living (BADL) by sociodemographic and health factors, depression, and multimorbidity, ELSI-Brazil, 2015–2016.

Variable	Difficulties in BADL		
	% (95% CI)	*p*-value	OR (95% CI)
Sex		0.151	
Female	14.0 (12.5; 15.7)		1
Male	12.7 (11.1; 14.4)		0.88 (0.75, 1.04)
Age group		<0.001	
50–59 years	12.1 (10.5; 14.0)		1
60–69 years	12.3 (10.5; 14.3)		1.01 (0.80; 1.27)
70–79 years	16.5 (13.9; 19.6)		1.43 (1.10; 1.85)
80 years and more	24.8 (20.1; 30.3)		2.38 (1.78, 3.19)
Marital status		0.021	
With a partner	12.6 (11.1; 14.2)		1
Without a partner	15.0 (13.3; 16.8)		1.22 (1.03; 1.45)
Color/race		0.016	
White	12.0 (10.1; 13.8)		1
Black	15.3 (12.5; 18.7)		1.35 (1.02; 1.79)
Brown	14.0 (12.6; 15.7)		1.22 (1.00; 1.47)
Yellow	18.3 (10.4; 30.2)		1.67 (0.83, 3.33)
Indigenous	20.7 (13.7; 30.2)		1.95 (1.12; 3.38)
Education		<0.001	
12 or more	6.9 (4.8; 10.0)		1
9–11 years	8.1 (6.5; 10.0)		1.17 (0.73, 1.88)
5–8 years	12.8 (10.6; 15.3)		1.97 (1.30; 2.99)
1–4 years	16.4 (14.6; 18.3)		2.63 (1.76, 3.91)
No formal education	19.9 (16.9; 23.2)		3.32 (2.11; 5.23)
Smoking		0.001	
Never smoked	11.4 (10.0; 12.9)		1
Former smoker	15.8 (13.9; 18.0)		1.46 (1.21; 1.74)
Smokes currently	13.5 (11.4; 15.8)		1.21 (1.00; 1.45)
Alcohol consumption		<0.001	
Does not consume	14.5 (13.2; 15.8)		1
Light/moderate consumption	9.5 (7.1; 12.6)		0.62 (0.46, 0.82)
Risk consumption	8.7 (6.6; 11.5)		0.56 (0.42; 0.74)
Consumption of fruits and vegetables		0.423	
Adequate	12.1 (9.1; 16.0)		1
Inadequate	13.5 (12.3; 14.9)		1.12 (0.83, 1.52)
Physical activity		<0.001	
Active	10.6 (9.3; 12.1)		1
Insufficiently active	19.5 (17.5; 21.7)		2.03 (1.73, 2.39)
Orientation in time		0.001	
All correct	8.6 (7.7; 9.5)		1
At least one incorrect	4.9 (4.1; 5.6)		1.45 (1.21; 1.74)
Verbal fluency		<0.001	
Lower tertile	6.2 (5.5; 7.0)		1
Intermediate tertile	3.5 (3.0; 4.0)		0.66 (0.55; 0.78)
Upper tertile	3.7 (3.0; 4.4)		0.53 (0.42, 0.67)
Memory score		<0.001	
Lower tertile	6.7 (5.9; 7.6)		1
Intermediate tertile	3.2 (2.7; 3.7)		0.71 (0.61, 0.83)
Upper tertile	3.5 (2.9; 4.1)		0.49 (0.38; 0.61)
Depressive symptoms		<0.001	
Absence (≤3 symptoms)	8.3 (7.0; 9.7)		1
Presence (≥4 symptoms)	23.7 (21.7; 25.8)		3.43 (2.91; 4.04)
Multimorbidity		<0.001	
Absence (≤1 disease)	8.1 (7.2; 9.2)		1
Presence (≥2 diseases)	5.3 (4.5; 6.0)		3.43 (2.92, 4.03)
Multimorbidity and depressive symptoms		<0.001	
No depressive symptoms and no multimorbidity	3.9 (3.2; 4.6)		1
Depressive symptoms	4.3 (3.7, 4.9)		3.03 (2.53, 3.63)
Multimorbidity	1.6 (1.2; 2.1)		2.94 (2.23, 3.87)
With depressive symptoms and multimorbidity	3.6 (3.1; 4.1)		8.36 (6.60; 10.59)

Concerning depressive symptoms, those with four or more symptoms had 3.43 times greater odds (OR: 3.43; 95% CI: 2.91; 4.04) of dependence for BADL than those with three or fewer depressive symptoms (Table 3). For those who reported multimorbidity, the odds of BADL difficulties were 3.43 times (OR: 3.43; 95% CI: 2.92; 4.03) higher than was for those who did not have multimorbidity. The combination of depressive symptoms and multimorbidity was associated with the presence of disability (OR: 8.36; 95% CI: 6.60; 10.59).

**TABLE 3:** Crude analysis adjusted for sociodemographic and health factors for difficulties in basic activities of daily living (BADL) with depression, multimorbidity, and their combination, ELSI-Brazil, 2015–2016.

Variable	BADL	
	Crude OR (95% CI)	Adjusted OR (95% CI)
Depressive symptoms		
Absence (≤3 symptoms)	1	1
Presence (≥4 symptoms)	3.43 (2.92,4.04)	3.22 (2.73, 3.80)
Multimorbidity		
Absence (≤1 disease)	1	1
Presence (≥2 diseases)	3.63 (2.99; 4.40)	3.57 (2.92, 4.37)
Multimorbidity and depressive symptoms		
No depressive symptoms and no multimorbidity	1	1
Depressive symptoms	3.03 (2.53,3.64)	2.79 (2.32, 3.35)
Multimorbidity	2.94 (2.23, 3.88)	2.72 (2.05, 3.61)
With depressive symptoms and multimorbidity	8.36 (6.60; 10.60)	7.74 (6.08; 9.86)

Adjusted by sex, age, color/race, marital status, education, smoking, alcohol consumption, consumption of fruits and vegetables, physical activity, and cognition.

The results of the associations, in the analysis adjusted by the sociodemographic and health factors, between BADL and depression, multimorbidity, and the groupings are presented in Table 3. The presence of depressive symptoms associated with the difficulty of performing BADL was 2.79 times (OR: 2.79; 95% CI: 2.32; 3.35) while with multimorbidity was 2.72 times (OR: 2.72; 95% CI: 2.05; 3.61) more than was for their peers. In the grouping of depression and multimorbidity, the odds of dependence on BADL were 7.74 times (OR: 7.74; 95% CI: 6.08; 9.86) higher than was for those who had no depressive symptoms and no multimorbidity.

### 3.2 IADL

In IADL, the difficulties were associated with those aged 70–79 years and 80 years or more, without a partner, indigenous, without a formal study, with 1–4 years of study, who were former smokers, physically inactive, with cognitive difficulty of temporal orientation, with depressive symptoms, and with multimorbidity (Table 4).

**TABLE 4:** Bivariate analysis of difficulties in instrumental activities of daily living (IADL) by sociodemographic and health factors, depression, and multimorbidity, ELSI-Brazil, 2015–2016.

Variable	IADL		
	% (95% CI)	*p*-value	OR (95% CI)
Sex		<0.001	
Female	51.9 (48.9; 55.0)		1
Male	37.3 (33.9; 40.8)		0.55 (0.48, 0.61)
Age group		<0.001	
50–59 years	38.9 (34.9; 43.2)		1
60–69 years	45.5 (42.7; 48.3)		1.30 (1.10, 1.55)
70–79 years	57.9 (54.0; 61.8)		2.16 (1.73, 2.69)
80 years and more	70.7 (64.5; 76.3)		3.78 (2.80, 5.11)
Marital status		<0.001	
With a partner	42.7 (39.2; 46.3)		1
Without a partner	49.7 (46.6; 52.8)		1.32 (1.17; 1.50)
Color/race		<0.001	
White	40.3 (36.6; 44.0)		1
Black	49.0 (44.7; 53.4)		1.42 (1.19; 1.70)
Brown	48.1 (45.0; 51.3)		1.37 (1.22, 1.55)
Yellow	50.8 (37.9; 63.6)		1.53 (0.90, 2.61)
Indigenous	57.0 (48.9; 64.7)		1.96 (1.38; 2.78)
Education		<0.001	
12 or more	26.7 (22.1; 31.9)		1
9–11 years	30.6 (27.1; 34.2)		1.20 (0.91, 1.59)
5–8 years	39.8 (35.8; 44.0)		1.81 (1.39, 2.36)
1–4 years	53.0 (49.7; 56.3)		3.09 (2.37, 4.03)
No formal education	71.5 (67.6; 75.1)		6.87 (5.11; 9.23)
Smoking		0.001
Never smoked	43.0 (39.7; 46.4)		1
Former smoker	47.9 (44.4; 51.4)		1.21 (1.08; 1.36)
Smokes currently	44.5 (40.3; 48.7)		1.06 (0.93, 1.19)
Alcohol consumption		<0.001	
Does not consume	48.9 (45.9; 52.0)		1
Light/moderate consumption	30.1 (26.5; 36.1)		0.47 (0.38, 0.57)
Risk consumption	28.8 (24.3; 33.8)		0.42 (0.34, 0.51)
Consumption of fruits and vegetables		0.022
Adequate	39.3 (33.1; 45.8)		1
Inadequate	45.7 (42.6; 48.8)		1.29 (1.03, 1.62)
Physical activity		<0.001	
Active	41.4 (38.3; 44.5)		1
Insufficiently active	53.4 (48.9; 57.9)		1.62 (1.39; 1.89)
Orientation in time		<0.001	
All correct	29.2 (27.5; 31.0)		1
At least one incorrect	15.9 (14.2; 17.8)		1.73 (1.53, 1.95)
Verbal fluency		<0.001	
Lower tertile	20.1 (18.0; 22.3)		1
Intermediate tertile	12.4 (11.4; 13.5)		0.61 (0.53; 0.70)
Upper tertile	12.6 (11.3; 13.9)		0.41 (0.35; 0.48)
Memory score		<0.001	
Lower tertile	21.6 (19.3; 24.0)		1
Intermediate tertile	11.1 (10.1; 12.1)		0.64 (0.56; 0.73)
Upper tertile	12.4 (11.3; 13.6)		0.39 (0.33; 0.45)
Depressive symptoms		<0.001	
Absence (≤3 symptoms)	35.9 (33.1; 38.9)		1
Presence (≥4 symptoms)	63.5 (59.3; 67.5)		3.09 (2.73, 3.51)
Multimorbidity		<0.0001
Absence (≤1 disease)	32.7 (30.2; 35.3)		1
Presence (≥2 diseases)	12.4 (11.1; 13.8)		2.78 (2.38, 3.25)
Multimorbidity and depressive symptoms		<0.001	
No depressive symptoms and no multimorbidity	19.0 (17.4; 20.7)		1
Depressive symptoms	13.7 (12.2; 15.3)		2.73 (2.37, 3.16)
Multimorbidity	4.9 (4.1; 5.8)		2.23 (1.79, 2.77)
With depressive symptoms and multimorbidity	7.4 (6.6; 8.4)		7.11 (5.71; 8.86)

Regarding depressive symptoms, the dependence on IADL among participants who presented four or more symptoms was 3.09 times (OR: 3.09; 95% CI: 2.73; 3.51) higher than was for those who presented three or less symptoms. Among the older adults with multimorbidity, the odds of dependence were 2.78 times (OR: 2.78; 95% CI: 2.38; 3.25) higher than was for those without multimorbidity. The odds of disability in IADL were 7.11 (95% CI: 5.71; 8.86) for those with depressive symptoms and multimorbidity when compared to those who did not have the outcomes.

The results of the adjusted analysis by sociodemographic and the health factors between IADL and depression, multimorbidity, and the combination of both are presented in Table 5. The odds of difficulty on IADL for those with depression were 2.36 (OR: 2.36; 95% CI: 2.02; 2.75) when compared to those without depressive symptoms. For multimorbidity, the disability in IADL was 2.04 times (OR: 2.04; 95% CI: 1.65; 2.53) higher than was for those without multimorbidity. In the group of depression and multimorbidity, the disability in IADL was 5.96 times (OR: 5.96; 95% CI: 4.76; 7.47) higher for people with depression and multimorbidity than was for those who had none.

**TABLE 5:** Crude analysis adjusted for sociodemographic and health factors for difficulties in instrumental activities of daily living (IADL) with depression, multimorbidity, and their combination, ELSI-Brazil, 2015–2016.

Variable	IADL	
	Crude OR (95% CI)	Adjusted OR (95% CI)
Depressive symptoms		
Absence (≤3 symptoms)	1	1
Presence (≥4 symptoms)	3.10 (2.73,3.51)	2.70 (2.36; 3.08)
Multimorbidity		
Absence (≤1 disease)	1	1
Presence (≥2 diseases)	3.03 (2.68,3.43)	2.82 (2.49; 3.19)
Multimorbidity and depressive symptoms		
No depressive symptoms and no multimorbidity	1	1
Depressive symptoms	2.74 (2.37,3.16)	2.36 (2.02, 2.75)
Multimorbidity	2.23 (1.79,2.77)	2.04 (1.65, 2.53)
With depressive symptoms and multimorbidity	7.11 (5.71,8.87)	5.96 (4.76; 7.47)

Adjusted by sex, age, color/race, marital status, education, smoking, alcohol consumption, consumption of fruits and vegetables, physical activity, and cognition.

## 4 Discussion

The increase in depressive symptoms and multimorbidity is associated with functional disabilities in Brazilian older adults, independently and combined in BADL and IADL. Depressive symptoms were present in 33.3% and multimorbidity in 18.9% of the older adults. Difficulties in one or more BADL and IADL were reported in 13.4% and 45.1%, respectively.

These results corroborate with studies of the older population in several countries (Garin et al., 2014; Arokiasamy et al., 2015; Quiñones et al., 2016; Agreli et al., 2017; Lee et al., 2018; Schmidt et al., 2020). In Brazil, among these, it was observed that the highest number of chronic diseases and depression was associated with a functional decline of the BADL and IADL (Agreli et al., 2017). Moreover, disabilities were associated with three multimorbidity patterns: cardiorespiratory (2.3% prevalence of multimorbidity) vascular metabolic (30.9% prevalence of multimorbidity), and mental musculoskeletal (12.9% prevalence of multimorbidity). The latter, which includes depression, presented disabilities in the BADL and IADL in 28.3% and 41.3% of the older adults, respectively (Schmidt et al., 2020). Most of the health problems experienced by older adults were due to chronic conditions. Some of them can be prevented by adopting healthy habits throughout life (Mundial de Saúde, 2015).

Healthy aging should be a part of everyone’s life. The intrinsic capacity of an individual, which is the combination of physical and mental capacities, is determined by several factors, such as physiological changes and the presence or absence of disease. Nevertheless, integrated and non-discriminatory care is required for healthy aging, especially for health promotion and disease prevention (World Health Organization, 2021). The combination of multimorbidity and depressive symptoms impairs healthy aging with physical and mental weakness, increasing the odds of disabilities.

The associated depressive symptoms and multimorbidity enhance the development of functional disabilities when performing BADL and IADL in Brazilian older adults. The preserved functional capacity results in a life with autonomy and independence, as it maintains the physical and mental abilities to perform BADL and IADL (Veras, 2009). Older adults are subject more to the development of disabilities not only due to their biological process of aging but also to social and affective issues, and the physical environment (Veras and Caldas, 2004). Thus, early identification of depression by professionals in primary healthcare is necessary due to the risk of developing other health problems and impaired mental health (Fässberg et al., 2016; Amaral et al., 2018; Silva et al., 2018).

The results presented in this study reinforce that the combination of depressive symptoms and multimorbidity worsens functionality, even adjusted for factors that may interfere with the outcomes. Chronic diseases in older adults potentiate the emergence of health problems and functional limitations. When there is depression added to other chronic diseases, the person also goes through a state of social isolation, a sedentary lifestyle, and cognitive and somatic changes, such as pain, as well as low self-esteem and abandonment of self-care.

The association in disability in BADL and IADL with the sociodemographic aspects has also been identified in other studies (Garin et al., 2014; Ćwirlej-Sozańska et al., 2019). The insertion of the family and partner in a person’s daily care allows them to perform their daily activities, maximizing the functional capacity (World Health Organization, 2005). As found in another Brazilian study in the Rio Grande do Sul State, the indigenous race showed an association with functional disabilities in IADL (Nunes et al., 2017a). Race/color is linked to socioeconomic status in Brazil as do lifestyle, housing, food, and education conditions. The habits of the indigenous people have given space to chronic non-communicable diseases, such as hypertension, cancer, and depression, due to changes mainly in lifestyle and diet (Garleno and Pontes, 2012).

Participants without a formal study had a higher prevalence of functional disabilities. It is observed that the longer the time of education, the greater the odds of the person maintaining autonomy to perform activities and a healthier lifestyle (Hoogendijk et al., 2008; Kagawa and Corrente, 2015; Agreli et al., 2017; Nunes et al., 2017b).

Among lifestyle habits, former smokers and physically inactive individuals were associated with increased disabilities in BADL and IADL, in the bivariate analysis. For the difficulties in IADL, there was also an association with low consumption of fruits and vegetables. A Brazilian study in the state of Paraíba found that older adults who did not practice physical activities developed more functional disabilities (Brito et al., 2016). Physical activity maintains good fitness, promotes health and disposition, and consequently decreases difficulties in performing daily activities. Physical activity is a therapeutic means to prevent and reduce physical and mental problems, and consequently, to greater independence in daily life (Ćwirlej-Sozańska et al., 2019).

The link between smoking cessation and functional disabilities is that former smokers usually abandon addiction by presenting health problems, which can also generate functional difficulties (Capilheira and Santos, 2006). The relationship between eating habits and difficulties in IADL may be due to low consumption of fruits and vegetables, contributing negatively to a good nutritional status and functional capacity. Older adults at risk of malnutrition have greater functional dependence than those with normal nutritional patterns (Lee and Tsai, 2012).

The results show the importance of preventing and investigating depression and multimorbidity. The early detection of risk factors that increase the prevalence of functional disabilities brings benefits to the person, their family, and the health system, to promote health with disease prevention. The active older adult, free of functional disabilities, becomes a protagonist in their lives, with autonomy to perform their daily activities and social participation with dignity and self-realization.

The limitations of the present study must be considered. First, the cross-sectional study prevents establishing causal relationships between exposure and outcome variables. Second, another point inherent to the study design is memory bias, which may be present in self-report questions, as participants may fail to inform some data, such as the number of existing chronic conditions. Above all, the study also has strengths. Data collection was performed based on validated scales recommended by the literature and applied by a trained team. It is also noteworthy that the data analyzed in this study are representative of the non-institutionalized Brazilian elderly population, as they were based on a national sample database with many participants.

## Data Availability

The data sets presented in this study can be found in online repositories. The names of the repository/repositories and accession number(s) can be found at https://elsi.cpqrr.fiocruz.br/data-access/.
